# The influence of casting techniques on the redisplacement risk of reduced distal radius fractures in adults

**DOI:** 10.1007/s00402-025-05910-z

**Published:** 2025-05-31

**Authors:** B. Barvelink, M. J. Kok, S. Smidt, K. F.C. Lakwijk, J. A.N. Verhaar, M. Reijman, J. W. Colaris

**Affiliations:** Erasmus MC, University Medical Center, Rotterdam, Netherlands

**Keywords:** Distal radius fracture, Cast immobilization, Non-operative treatment, Redisplacement, Cast index

## Abstract

**Introduction:**

Successfully reduced distal radius fractures (DRFs) often redisplace while casted. Poor cast moulding might be a risk factor for redisplacement of DRFs. This study aims to assess whether cast moulding quality, as determined by casting indices, impact the risk of redisplacement. Also, we assessed the influence of the cast applicant and the material used on the redisplacement risk.

**Materials and methods:**

We retrospectively reviewed cases from a prospective cohort (trial registration NL8311). We included 172 adequately reduced and circumferentially casted DRFs with a complete two-week radiographic follow-up. Fracture alignment was measured on all radiographs (trauma, post-reduction and follow-up) in accordance with the Dutch guideline for DRFs. When unacceptably aligned after 2 weeks, the DRF was labelled as redisplaced. Cast moulding quality was measured using the Three Point Index (TPI), Cast Index (CI) and Gap Index (GI). A TPI > 0.8, CI > 0.7 and GI > 0.15 implicates poor cast moulding. Multivariable logistic regression was used to examine the influence of cast moulding quality, cast applicant and casting material on the redisplacement risk. We corrected for patient age, intra-articular involvement, the degree of radial inclination and radial shortening.

**Results:**

Redisplacement occurred in 40% of DRFs. The mean index scores were poor (TPI 0.94, CI 0.85, GI 0.22), indicating generally suboptimal cast moulding quality. None of the cast indices were significantly associated to redisplacement (OR [95% CI]: TPI 1.2 [0.6 to 2.5], CI 2.4 [0.7 to 15.7], GI 1.6 [0.7 to 4.0]). DRFs casted by nurse practitioners had significantly lower odds of redisplacement compared to those casted by emergency room nurses. Type of casting (synthetic versus plaster of Paris) was not associated with redisplacement.

**Conclusions:**

Cast moulding quality, measured using cast indices, is not associated with redisplacement of reduced DRFs. Casts applied by nurse practitioners redisplaced significantly less often.

**Level of Evidence:**

Therapeutic Studies level III

**Supplementary Information:**

The online version contains supplementary material available at 10.1007/s00402-025-05910-z.

## Introduction

A significant proportion—32 to 64%—of reduced distal radius fractures (DRFs) in adults will redisplace during non-operative treatment [1–4]. Preventing redisplacement would be ideal as it would lead to less surgical interventions with benefit for both patient and healthcare costs. In the field of paediatric fractures, it is suggested that cast moulding quality affects the redisplacement risk [5–8]. This association is only scarcely investigated in the adult population. Three studies in adults have been published on this topic, reporting contradictory conclusions [9–11]. Cast moulding quality can be described to as the quality of plaster moulding and padding in a way it provides best support to the fracture zone. Several cast indices are described to measure cast moulding quality, including the Cast Index (CI) [12], the Gap Index (GI) [5] and the Three-Point Index (TPI) [6]. The TPI has shown highest specificity and sensitivity in predicting redisplacement in both paediatric and adult forearm fractures [6, 11]. In a retrospective cohort, a promising sensitivity of 96% and specificity of 96% was found for the TPI [11].

In addition to cast moulding quality, the influence of the occupation of the healthcare provider applying the plaster—casting technician, emergency room nurse or nurse practitioner—on the redisplacement risk is unknown. Also, different types of casting material are available these days. The influence of plaster of Paris versus synthetic casting on redisplacement has not yet been investigated.

This study aims to investigate if cast moulding quality, as determined by the TPI, CI and GI, is associated with redisplacement in a prospective cohort of adult patients with adequately reduced DRFs. In addition, we investigate whether the cast applicant (casting technician, emergency room nurse or nurse practitioner) and the choice of casting material (plaster of Paris or synthetic cast) are of influence on the redisplacement risk.

## Methods

### Study design

This observational follow-up study retrospectively reviews prospectively collected radiographs from a subset of patients who participated in the CAST study, a pragmatic multicentre cluster-randomised controlled trial (METC 2019 − 0528, registered at ClinicalTrials.gov [NL8311]) [4, 13]. Patients were included in ten hospitals in the Netherlands from May 2020 to November 2021. In the CAST study, a total of 752 patients (age ≥ 18 years) with reduced DRFs that started conservative treatment were included and randomized between immobilization using plaster splinting and circumferential casting. Exclusion criteria comprised: both-bone fractures (a solitary ulnar styloid process fracture was accepted), trauma patients with an injury severity score above 16, concomitant injury to the ipsilateral extremity and the inability to complete questionnaires due to a language barrier or cognitive impairment. Written informed consent was obtained for every patient.

### Participants

For the current study, cases from the CAST study cohort were selected with an acceptable fracture alignment after reduction, conform the Dutch guideline for DRFs, published in 2010 [14]. The Dutch Guideline for DRFs stated that a DRF is unacceptable aligned if one or more of the following criteria are met when measuring alignment on the radiographs: ≥ 15° dorsal angulation, ≥ 20°, palmar angulation, ≤ 15° radial inclination, ≥ 3 mm radial shortening and ≥ 2 mm intra-articular step-off or gap. We only included patients that received a circumferential cast after reduction in this manuscript since casting indices are not applicable, neither measurable on splinted fractures. In all included cases, a below-elbow circumferential cast was applied with the hand in neutral position, conform instructions that were available in all participating hospitals by means of instructional videos and posters [13]. Circumferential casts could be made with either plaster of Paris or synthetic material (e.g. fiberglass), based on availability of materials or preference of the cast applicant. Radiographic follow-up took place at one week, two week and five weeks post-inury. If radiographic follow-up was incomplete during the first two treatment weeks, the patient was excluded. In case of severe cast complaints or insufficient casting, casting technicians were free to make cast alterations during follow-up to secure adequate fracture support.

### Primary outcome

The primary outcome in this study is the redisplacement incidence of DRFs. For defining redisplacement, fracture alignment was measured on all radiographs (postero-anterior [PA] and lateral [LAT] view at trauma, post-reduction, 1 week and 2 week follow-up). Measurements were executed by two trained researchers (BB and SS) conform the measurement guidelines carried out by Medoff et al. (2005) [15]. Inter- and intra-observer reliability were calculated, using intraclass correlation coefficients (ICC, two-way mixed and absolute agreement). Intra-observer reliability for gap/step-off measurements on lateral radiographs was moderate (ICC 0.56). The ICC’s for all other alignment measurements were excellent (ICC of 0.84 to 0.99). The endpoint for redisplacement was chosen at two weeks of treatment, since the primary applied cast is often replaced after two weeks. A longer follow-up could therefore introduce bias because of fracture redisplacement initiated by cast replacement or alignment changes influenced by a second attempt to closed reduction.

Redisplacement is described and analysed in two ways. Firstly, as the loss of acceptable alignment conform the Dutch guideline. Fractures that were correctly aligned on post-reduction radiographs, but had an unacceptable alignment after two weeks, were labelled as redisplaced. When correct alignment was maintained during two weeks, the fracture was labelled as non-displaced. Secondly, redisplacement is described in terms of fracture migration. DRFs most often loose threshold alignment because of progressive angulation or decreasing inclination over time [10]. Migration is defined as the absolute amount of displacement, in degrees of angulation and inclination. For example; one degree of dorsal angulation post-reduction could redisplace to 14 degrees of dorsal angulation after two weeks. The fracture remains stable conform the Dutch guideline, but could be considered as unstable because of the thirteen degrees of fracture migration.

### Potential risk factors for redisplacement

**Fig. 1:**
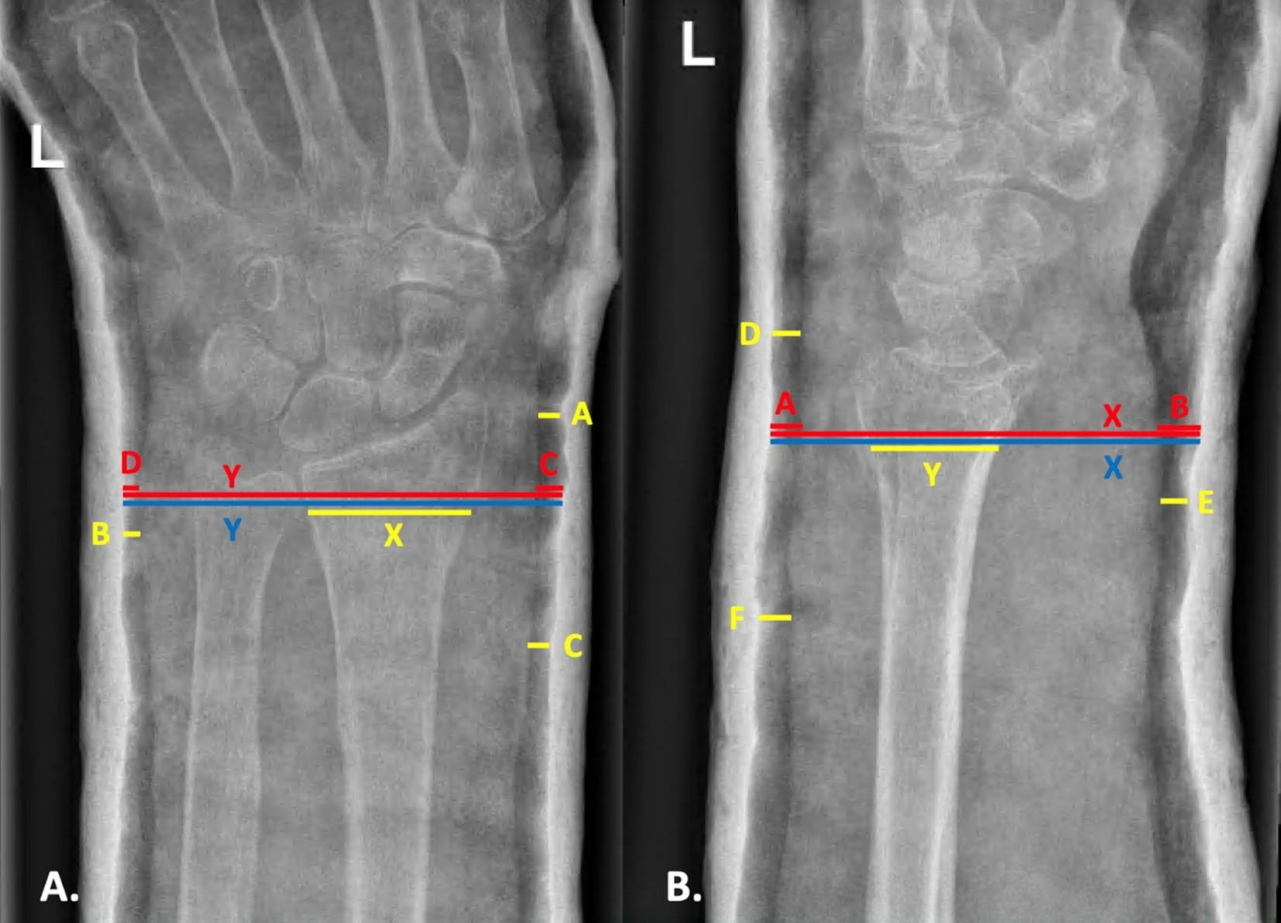
**A.** and **B.** Cast indices measurements shown on postero-anterior (**A**) and lateral (**B**) radiographs

Cast moulding quality was evaluated using three different cast indices, namely the TPI, CI and GI. These indices are based on specific spaces and distances in different cast regions that are considered important to optimally stabilise the fracture. Indices were measured on the PA and LAT radiographs taken after reduction and cast application. The measurements and indices formulas are shown in Fig. 1A and B, and measured as described in previous literature [5, 6, 12]. The TPI is calculated by adding three distances between the cast and the skin measured on the PA radiograph, divided by the contact length of the fracture. The same is done on the LAT radiograph. The sum of these two calculations constitutes the TPI. The CI is a measure of the inside diameter of the plaster on the LAT-radiograph as a ratio to the diameter on the PA-radiograph at the fracture site [12]. The GI is a measure of the space between the plaster and the skin, measured as a ratio to the inside diameter of the plaster. This is measured at the fracture site in both the PA and LAT radiographs [5]. The cut-off values for the indices are 0.8 for the TPI, 0.7 for the CI and 0.15 for the GI. A score up to the cut-off value represents a well-moulded cast whereas a score above the cut-off value represents a poorly moulded cast. Index measurements were performed by one trained researcher (MK) and a subset was measured by another trained researcher (KL) for a reproducibility analysis. The inter- and intra-observer reliability of the TPI, CI and GI were excellent, with ICC’s ranging between 0.91 and 1.0.

Yellow represents the Three-Point Index. A: The narrowest space around the radiocarpal or proximal carpal joint on the radial side; B: The narrowest space on the ulnar side, within 1 cm of the fracture line; C: The narrowest space on the radial side, 3 to 7 cm proximal of the fracture site; X: The contact length of the fracture; D: The narrowest gap on the dorsal side, around the radiocarpal or proximal carpal joint; E: The narrowest gap on the palmar side, within 1 cm of the fracture line; F: The narrowest gap on the dorsal side, within 3 to 7 cm proximal to the fracture site; NB. In less common palmar angulated fractures, D and F are measured on the palmar side and E on the dorsal side. Y: The contact length of the fracture. TPI formula: [(A + B + C)/X] + [(D + E + F)/Y].

Red represents the Gap Index. C: The gap between plaster and skin at the fracture zone on the radial side; D: The gap between plaster and skin at the fracture zone on the ulnar side; Y: The inside cast diameter at the fracture zone; A: The gap between plaster and skin at the fracture zone on the dorsal side; B: The gap between plaster and skin at the fracture zone on the palmar side; X: The inside cast diameter at the fracture line. GI formula: (A + B)/X + (C + D)/Y.

Blue represents the Cast Index. Y: The inside cast diameter at the fracture zone; X: The inside cast diameter at the fracture zone. CI formula: X/Y.

The occupation of healthcare provider applying the cast and the type of material used (plaster of Paris or synthetic casting) were denoted in a questionnaire filled out by the healthcare professional treating the patient at the time of inclusion. The CAST study was a pragmatic trial, and therefore the choice for cast applicant or material used was unconstrained.

### Statistical analyses

Statistical analyses were performed using IBM SPSS version 28.0.1.0. P-values ≤ 0.05 are considered significant. Whether cast moulding quality (cast index above or beneath cut-off value), cast applicant or casting type are related to redisplacement (dependent variable) have been tested with multivariable logistic regression analyses. We corrected for potential confounding factors namely age and the following fracture characteristics at trauma: AO classification, intra-articular fracture involvement, the degree of radial inclination and radial shortening. Univariate logistic regression was used to select potential confounding patient- and fracture related factors. In case a relationship existed between the variable and redisplacement (*p* < 0.2), the variable was added in the multivariable logistic regression model. The sensitivity, specificity, positive predictive value, negative predictive value of the indices were calculated. To test whether casting type or type of cast applicant predicts migration outcome, we used multiple linear regression analysis. We corrected for the same potential confounders as mentioned for the logistic regression analyses. One-way ANOVA was used to compare cast indices outcomes amongst the cast applicants.

## Results

**Fig. 2:**
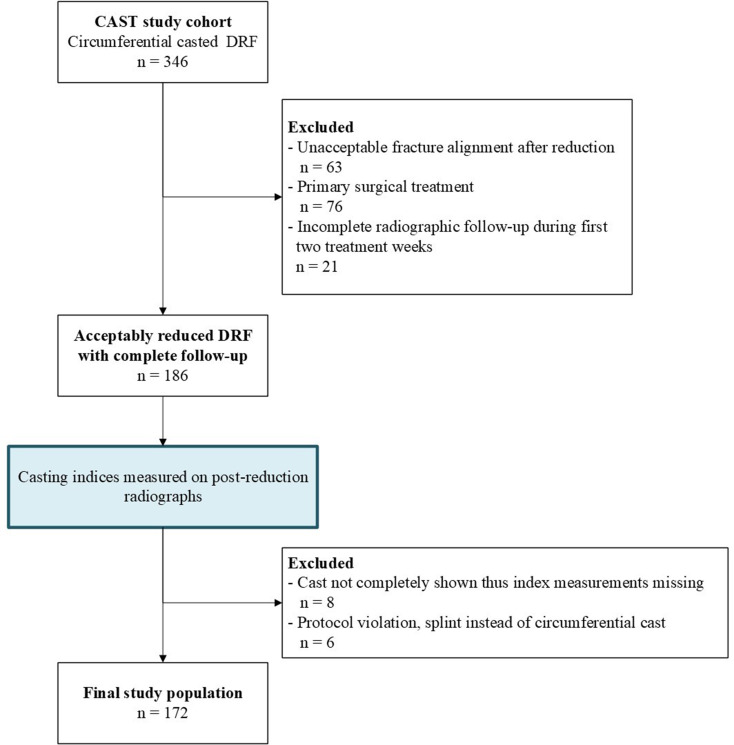
Flowchart of study population

The inclusion criteria were met by 186 patients of which 172 were used in the final analyses (Fig. 2). Eight patients were excluded from the final analyses because the cast was not completely visible on radiographs and 6 patients did not receive the allocated circumferential cast but were treated with a splint. Patient and fracture characteristics of the final study population are shown in Table 1.

**Table 1:** Patient and fracture characteristics of the study population

	Study population *n* = 172		
*Patient characteristics*			
Female, n (%)	142 (83)		
Age, years (SD, min-max)	62 (16.4, 18–91)		
BMI, kg/m^2^ (SD, min-max)	24 (3.8, 16–40)		
*Fracture characteristics*			
Dominant wrist affected, n (%)	87 (51)		
Styloid ulnae fracture, n (%)	97 (56)		
Intra-articular, n (%)	80 (47)		
AO classification, n (%) A type B type C type	92 (53) 13 (8) 67 (39)		
	Before reduction	Post reduction	At two weeks (*n* = 142*)
Dorsal angulation, n (%)	160 (93)	93 (54)	58 (70)
Angulation, ° (SD, min-max)	21 (10.4, 0–59)	5 (4.1, 0–19)	8 (6.5, 0–37)
Radial inclination, ° (SD, min-max)	17 (6.4, -5-28)	22 (3.6, 16–34)	19 (4.8, 7–30)
Radial shortening (yes)	85 (49)	27 (16)	55 (32)
- if yes: Severity of radial shortening in mm (SD, min-max)	3.0 (2.4, 0–14)	1.3 (1.2, 0–6)	1.1 (1.2, 0–5)

If not noted differently, information is presented as mean with standard deviation and range between parentheses. N: number of cases, BMI: body mass index

*in 30 cases patients were treated surgically after redisplacement occurred at 1 week. Therefore no control-radiograph at two weeks is available

### Influence of cast moulding quality on the redisplacement risk

Fracture redisplacement as defined by the guideline occurred in 40% of DRFs (*n* = 76) within the first two weeks of immobilization. Cast indices outcomes are shown in Table 2. All three indices scored a mean index outcome above the threshold score, referring to poor cast moulding. There was no association found between casting indices and whether a fracture would redisplace or not conform the guideline (Table 3). Sensitivity, specificity, negative predictive value and positive predicting values of all indices are reported in the supplementary appendix, Table S1. During the first two weeks of cast immobilization, fractures showed a mean change in palmar tilt (migration in angulation) of 7.0 degrees (SD 6.4),and a mean loss of inclination (inclination migration) of 3.1 degrees (SD 3.6). These changes in palmar tilt and loss of inclination were not related to cast indices outcomes (details in Supplementary Appendix, Table S2). In 25 cases (13%), a cast replacement took place within two weeks. The redisplacement incidence, as well as mean cast indices outcomes were not different from the total study cohort (details in Supplementary Appendix, Table S3).

**Table 2:** Index outcome distribution

			Poor cast moulding conform index	
	Index threshold^a^	Mean (SD)	Non-displaced *n* = 103 n (%)	Displaced *n* = 69 n (%)
Three point index	0.8	0.94 (0.36)	58 (56)	41 (59)
Casting index	0.7	0.85 (0.06)	99 (96)	67 (97)
Gap index	0.15	0.22 (0.07)	82 (80)	55 (80)

^a^An index score above the threshold value refers to poor cast moulding quality

**Table 3:** Association of casting indices, type of cast applicant and type of casting material on the occurrence of fracture redisplacement

	Redisplacement incidence (%)	Odds ratio (95% CI)
Three Point Index > 0.8	59	1.2 (0.61 to 2.55)
Cast index > 0.7	97	2.4 (0.38 to 15.75)
Gap index > 0.15	80	1.6 (0.68 to 4.01)
Cast applicant		
Emergency room nurse	52	Reference
Nurse practitioner	22	0.2 (0.09 to 0.54)*
Casting technician	39	0.5 (0.20 to 1.19)
Casting material		
Plaster of Paris	45	Reference
Synthetic fiber	31	0.56 (0.27 to 1.14)

Multivariable logistic regression analyses was used, corrected for patient age, AO classification, fracture inclination, radial shortening, intra-articular fracture involvement. *P < 0.01

### Influence of cast applicant on the redisplacement risk

In this cohort, casts were applied by five types of healthcare providers, namely emergency room (ER) nurses (*n* = 83), nurse practitioners (*n* = 50), casting technicians (*n* = 36), an ER specialist (*n* = 1) and a resident in orthopaedic surgery (*n* = 1). As shown in Table 3, the redisplacement risk was lowest in casts applied by nurse practitioners and casting technicians. The odds of a fracture to redisplace was significantly lower for fractures that were casted by nurse practitioners compared to ER nurses (odds ratio [OR] 0.2, 95% CI 0.09 to 0.54). However, the cast applicant was not significantly associated with migration outcomes (Table 4). Cast indices did not significantly differ between cast applicants. All mean index scores reached above the cut-off values (details in Supplementary Appendix, Table S4).

**Table 4:** Association of casting material and cast applicant on fracture migration

	Angulation migration	Coefficient ß	Standard Error	*p*-value
*Casting material*				
Plaster of Paris	7.5 (6.9, 0–34)			Reference
Synthetic fiber	6.8 (5.7, 0–33)	-0.98	1.04	0.35
*Cast applicant*				
Emergency room nurse	7.3 (5.8, 0–21)			Reference
Nurse practitioner	7.3 (7.8, 0–34)	-0.74	1.17	0.53
Casting technician	7.0 (6.7 0–33)	-1.13	1.29	0.38
	Inclination migration	Coefficient ß	Standard Error	p-value
*Casting material*				
Plaster of Paris	3.3 (3.7, -15-17)			Reference
Synthetic fiber	2.6 (3.1, -4-10)	-0.44	0.52	0.40
*Cast applicant*				
Emergency room nurse	3.7 (4.0, -5-17)			Reference
Nurse practitioner	2.8 (2.9, -3-10)	-0.57	0.58	0.33
Casting technician	2.3 (3.0, -4-9))	-1.26	0.64	0.05

Migration outcomes are given as mean degrees with standard deviation and range between brackets. Multivariable linear regression analyses was used, corrected for patient age, AO classification, fracture inclination, radial shortening and intra-articular fracture involvement

*Influence of cast material on the redisplacement risk*Two types of cast material were used in this cohort to immobilize the fracture, namely plaster of Paris (*n* = 113 [66%]) and synthetic casting (*n* = 59 [34%]). DRFs immobilized with synthetic casting redisplaced less often compared to plaster of Paris (31% vs. 45%) but this association was not significant (OR 0.56, 95% CI 0.27 to 1.15, Table 3). Concerning migration outcomes, mean degrees of migration were lower in synthetic casts compared to plaster of Paris. The linear regression analysis however concludes that the material used for casting does not explain the variation in migration (Table 4).

## Discussion

This retrospective study, utilizing prospective collected data, did not demonstrate any association between the cast moulding quality and the incidence of fracture redisplacement in adult DRFs. The predictive performances of the three tested indices (TPI, CI and GI) were poor. The cast indices scores were not related to the extent of migration in angulation or inclination during follow-up. This indicates that a poor cast moulding is not a risk factor for redisplacement. As for secondary outcomes, we found that casts applied by nurse practitioners resulted in less migration and redisplacement than casts applied by ER nurses. We did not identify any correlation between various cast materials and the occurrence of fracture redisplacement.

The rationale for this study was the absence of conclusive evidence regarding the influence of casting moulding quality on the redisplacement risk in adults. Only three studies have been published reporting on casting indices in adults [9–11]. Remarkably, Alemdaroğlu et al. found very high predictive performances of the TPI, implicating that insufficient cast moulding is the most important risk factor for redisplacement. However, we were unable to replicate these results. For example, we calculated a specificity of 44% compared to 96% in their study. A difference in redisplacement criteria could play a role in this. They defined a fracture as redisplaced when there was an increase of 10 degrees of dorsal or palmar angulation. However, the results of our analyses on angulation migration did not reveal any significant association either. We consequently doubt the value of cast indices in the adult population. When casting indices would adequately describe cast moulding quality, we would expect cast indices to be at least beneath threshold values when applied by casting technicians. Since the predictive performances of all three indices were poor in our cohort, we propose that casting indices are not useful as a tool to measure cast moulding quality in adult DRFs. Two studies support this opinion. Siddiqui et al. performed a retrospective study examining the TPI in 54 adults and they concluded that the TPI could not predict redisplacement [9]. Mimura et al. recently concluded that the gap index is also not associated with redisplacement [10]. Unfortunately, this research question was a small sub question in their study and therefore the used methodology has not been described in detail. Our study shows conclusively that cast moulding quality as measured by casting indices, is not associated to redisplacement.

The overall incidence of fracture redisplacement was 40% in this cohort. This incidence is comparable with reported incidences in previous prospective trials [4, 16–19]. We deliberately chose not to elaborate on the treatment of these fractures beyond two weeks, as the focus of this manuscript is solely on the impact of casting techniques on radiographic alignment. A detailed flowchart of follow-up decisions is provided in the main outcome paper of the CAST study [4],. Despite adequate initial reduction, a high rate of redisplacement is well documented and continues to be a subject of ongoing discussion. Nevertheless, patient-reported outcomes and clinical results following non-operative management are generally favorable, even when radiographic alignment is not restored or maintained [4, 16, 20]. Consequently, achieving restored radiographic alignment may not be essential for a satisfactory outcome. Particularly in individuals with lower functional demands, non-operative treatment should therefore be considered as a treatment option.

As for the secondary outcomes, this current study is, to the best of our knowledge, the first to analyse whether redisplacement risks and casting indices differ between different specializations of cast applicants and between casts of different materials. Abson et al. studied if casting quality in paediatric fractures varied amongst surgeons with different levels of experience [21]. No significant difference was found. In our cohort, the redisplacement risk was lower in casts applied by nurse practitioners and casting technicians, compared to casts applied by ER nurses. The current results might imply that the experience of the cast applicant is of value for the redisplacement risk. It should be considered that post-reduction alignment might be a confounding factor. In the Netherlands, closed reductions are predominantly carried out by young and relatively inexperienced junior doctors. Specialized ER nurse practitioners and casting technicians typically possess greater experience with reducing fractures. Consequently, it may be plausible that their reductions more often result in a stable and (close to) anatomical fracture alignment. With regard to the influence of casting material, although not statistically significant, synthetically casted DRFs redisplaced less often in comparison to fractures treated with plaster of Paris. The redisplacement incidence was 14% lower. It could be argued that choice for a certain casting type might be influenced by patient- and fracture characteristics. An explorative analyses on this matter, shown in the supplementary appendix, Table S5 revealed that groups were comparable.

This study has certain limitations. First, while our overall group size is larger than that of related previous studies, the subgroup sizes were relatively small and therefore the results of subgroup analyses should be interpreted with caution. In our analyses, we adjusted for known factors that influence the redisplacement risk to minimize the risk of bias. Despite thorough checks with univariate logistic regression, other confounders may exist. For example, we did not study fracture comminution as it is not included in the national guideline and because it is difficult to measure the extend objectively on radiographs [22]. Lastly, in 21 cases (12.2%), C and/or F regions of the TPI were not completely visible on radiographs. In theory the TPI could therefore be underestimated in these cases. The analysis was repeated with the exclusion of these cases which did not alter the outcome. Therefore, all cases were retained and included in the analyses.

The strength of the present study lies in the detailed nature of the prospectively gathered data and its multicentre design. Hereby, a good reflection of the diversity in fractures and casts in daily practice is provided. Second, to the best of our knowledge this is the first study to compare the predictive performances of different casting indices in adults and to investigate the association between different cast applicants and cast materials used. Third, we decided to measure redisplacement by measuring fracture migration, in addition to adhering to established redisplacement criteria. Given the potential variations in redisplacement guidelines across regions and with changing standards over time, utilizing absolute measures of migration provides a more robust approach.

This study concludes that cast moulding quality as measured using the TPI, CI and GI does not significantly influence the risk of redisplacement in reduced adult DRFs. The utility of cast indices in describing cast moulding quality is doubted. However, an association was found between cast applicants and the redisplacement risk, in which DRFs casted by nurse practitioners had the smallest redisplacement risk. This suggests that experience in cast application decreases the redisplacement risk.

## Electronic supplementary material

Below is the link to the electronic supplementary material.

Supplementary Material 1

## Data Availability

No datasets were generated or analysed during the current study.
