# Correction: The influence of casting techniques on the redisplacement risk of reduced distal radius fractures in adults

**DOI:** 10.1007/s00402-025-06071-9

**Published:** 2025-10-10

**Authors:** B. Barvelink, M. J. Kok, S. Smidt, K. F.C. Lakwijk, J. A.N. Verhaar, M. Reijman, J. W. Colaris, The CAST study group, Flip van Beek, Marna G. Bouwhuis, Milko M. M. Bruijninckx, Alexander P. A. Greeven, Taco Gosens, Marike C. Kokke, Gerald A. Kraan, Michiel Leijnen, Merel van Loon, Daphne A. van Rijssel, Merel van Loon, Daphne A. van Rijssel, Niels W. L. Schep, Lenneke Scholtens, Ninka Slebioda, Ninka Slebioda, Mathieu M. E. Wijffels, Peer van der Zwaal, Egon Zwets

**Affiliations:** Erasmus MC, University Medical Center, Rotterdam, Netherlands

**Correction: Archives of Orthopaedic and Trauma Surgery (2025) 145:326**

10.1007/s00402-025-05910-z

In the original version of this article, the ‘CAST study group’ was missing in the author group

Author group, which previously read as

B. Barvelink^1^ · M. J. Kok^1^ · S. Smidt^1^ · K. F.C. Lakwijk^1^ · J. A.N. Verhaar^1^ · M. Reijman^1^ · J. W. Colaris^1^

but should have read

B. Barvelink^1^ · M. J. Kok^1^ · S. Smidt^1^ · K. F.C. Lakwijk^1^ · J. A.N. Verhaar^1^ · M. Reijman^1^ · J. W. Colaris^1^ · The CAST study group

**Acknowledgement**

The CAST study group consists of the following members:

 Flip van Beek^1^, Marna G Bouwhuis^2^, Milko M M Bruijninckx^3^, Alexander P A Greeven^4^, Taco Gosens^5^, Marike C Kokke^6^, Gerald A Kraan^7^, Michiel Leijnen^8^, Merel van Loon^9^, Daphne A van Rijssel^10^, Niels W L Schep^11^, Lenneke Scholtens^12^, Ninka Slebioda^13^, Mathieu M E Wijffels^14^, Peer van der Zwaal^15^, Egon Zwets^16^

^1^Department of Trauma Surgery, Franciscus Hospital, Schiedam and Rotterdam, the Netherlands. ^2^Department of Emergency Medicine, Erasmus MC, University Medical Centre Rotterdam, the Netherlands. ^3^Department of Trauma Surgery, IJsselland Hospital, Capelle aan den IJssel, the Netherlands. ^4^Department of Trauma Surgery, Haga Teaching Hospital, The Hague, the Netherland. ^5^Department of Orthopaedic Surgery, Elisabeth-Tweesteden Hospital, Tilburg, the Netherlands. ^6^Department of Trauma Surgery, St. Antonius Hospital, Utrecht and Nieuwegein, the Netherlands. ^7^Department of Orthopaedic Surgery, Reinier de Graaf Gasthuis, Delft, the Netherlands. ^8^Department of Trauma Surgery, Alrijne Hospital, Leiderdorp, the Netherlands. ^9^Department of Emergency Medicine, Haaglanden Medical Centre, The Hague, the Netherlands. ^10^Department of Emergency Medicine, Reinier de Graaf Gasthuis, Delft, the Netherlands. ^11^Department of Trauma Surgery, Maasstad Hospital, Rotterdam, the Netherlands. ^12^Department of Emergency Medicine, Haga Hospital, The Hague, the Netherlands. ^13^Department of Orthopaedics and Sports Medicine, Erasmus MC, University Medical Centre Rotterdam, the Netherlands. ^14^Trauma Research Unit, Department of Surgery, Erasmus MC, University Medical Centre Rotterdam, the Netherlands. ^15^Department of Orthopaedic Surgery, Haaglanden Medical Centre, the Hague, the Netherlands. ^16^Department of Emergency Medicine, Franciscus Hospital, Schiedam and Rotterdam, the Netherlands

