# Supplementary material for: The influence of casting techniques on the redisplacement risk of reduced distal radius fractures in adults
Source: Arch Orthop Trauma Surg. 2025 May 31;145(1):326. doi: 10.1007/s00402-025-05910-z (PMC12126326; doi:10.1007/s00402-025-05910-z)
Supplement: Supplementary file 1 — Supplementary Material 1 [file 402_2025_5910_MOESM1_ESM.docx]

**Supplementary Appendix**

**Table S1.** Sensitivity, specificity, negative predictive value and positive predictive value of all three indices.

|  | Three point index | Casting index | Gap index |
| --- | --- | --- | --- |
| Sensitivity | 59.4% | 97.1% | 79.7% |
| Specificity | 43.7% | 3.9% | 20.4% |
| Positive predictive value | 41.4% | 40.4% | 40.1% |
| Negative predictive value | 61.6% | 66.7% | 60.0% |

Predictive performances of index outcome above threshold scores for predicting redisplacement.

**Table S2.** Association of casting indices with fracture migration.

|  | Three point index | | | Cast index | | | Gap index | | |
| --- | --- | --- | --- | --- | --- | --- | --- | --- | --- |
|  | ‘good’  (≤ 0.8) | ‘poor’  (> 0.8) | P-value | ‘good’  (≤ 0.7) | ‘poor’  (> 0.7) | P-value | ‘good’  (≤ 0.15) | ‘poor’  (> 0.15) | P-value |
| Migration in angulation, ° | 6.7 (5.5) | 7.4 (7.0) | 0.98 | 3.8 (2.7) | 7.1 (6.5) | 0.22 | 7.6 (5.1) | 7.0 (6.7) | 0.18 |
| Migration in inclination, ° | 3.4 (3.1) | 2.9 (3.9) | 0.33 | 2.1 (1.2) | 3.1 (3.6) | 0.40 | 3.6 (3.7) | 3.0 (3.5) | 0.22 |

Results are presented as means with standard deviation between brackets.

**Explorative analysis early cast replacements.**

In 25 cases (13%), the circumferential cast was replaced by a new circumferential cast within two weeks. In 4 cases, the cast replacement took place within one week because of cast complaints. In the other cases, the cast was replaced at the scheduled follow-up visit at the casting room one week after injury. The reasons for these cast replacements was not further specified.

In this cohort, fracture redisplacement occurred in 10 cases (40%). This is consistent with the complete cohort. Cast index outcomes are shown in table 2. Mean index scores and their relation to cast redisplacement are not different from the complete study cohort.

**Table S3.** Index outcome distribution.

|  |  |  | Poor cast moulding  conform index | |
| --- | --- | --- | --- | --- |
|  | Index threshold^a^ | Mean (SD) | Non-displaced  n = 15  n (%) | Displaced  n = 10 n (%) |
| Three point index | 0.8 | 1.13 (0.33) | 9 (75) | 6 (67) |
| Casting index | 0.7 | 0.85 (0.04) | 12 (100) | 9 (100) |
| Gap index | 0.15 | 0.25 (0.07) | 11 (92) | 7 (78) |

^a^An index score above the threshold value refers to poor cast moulding quality.

**Table S4.** Association of healthcare provider with fracture migration.

|  | n | Three point index | Cast index | Gap index |
| --- | --- | --- | --- | --- |
| ER nurse | 83 | 0.97 (0.36) | 0.85 (0.05) | 0.22 (0.08) |
| Nurse practitioner | 50 | 0.85 (0.33) | 0.86 (0.06) | 0.20 (0.06) |
| Casting technician | 36 | 0.99 (0.36) | 0.85 (0.08) | 0.23 (0.08) |
| P value |  | 0.12 | 0.38 | 0.12 |

Results are presented as means with standard deviation between brackets. ER: Emergency Room, n: number of patients

**Explorative analyses casting type**

No significant differences were seen concerning patient- and fracture characteristics between both casting types (see S4). Synthetic casting is most often applied by casting technicians and nurse practitioners. ED nurses use synthetic casting in 7% of cases, whilst casting technicians and nurse practitioners use synthetic casting more frequently, with 69% and 54% respectively.

**Table S5**. Patient- and fracture characteristics for both casting types.

|  | Synthetic fiber  n=59 | Plaster of Paris  n=113 | P value |
| --- | --- | --- | --- |
| Patient characteristics |  |  |  |
| Female, n (%) | 50 (85) | 93 (82) | 0.30 |
| Age, years | 64 (14) | 62 (17) | 0.18 |
| BMI, kg/m^2^ | 24 (4) | 24 (4) | 0.38 |
|  |  |  |  |
| Fracture characteristics |  |  |  |
| Dominant side affected, n (%) | 32 (54) | 55 (49) | 0.48 |
| Angulation, ° | 21 (10) | 21 (11) | 0.64 |
| Inclination, ° | 18 (5) | 16 (7) | 0.09 |
| Styloid Ulnae fracture, n (%) | 36 (61) | 61 (54) | 0.38 |
| Radial shortening, n (%) | 34 (58) | 51 (45) | 0.16 |
| Intra-articular, n (%) | 26 (44) | 54 (48) | 0.64 |

If not noted differently, information is presented as mean with standard deviation between parentheses. N: number of patients, BMI: body mass index.
